# Downregulation of long non-coding RNA LET predicts poor prognosis and increases Notch signaling in non-small cell lung cancer

**DOI:** 10.18632/oncotarget.23452

**Published:** 2017-12-19

**Authors:** Shengwen Li, Hui Zhao, Jianqiang Li, Aizheng Zhang, Haibin Wang

**Affiliations:** ^1^ Shanxi Medical University, Taiyuan, Shanxi 030001, China; ^2^ Department of Respiratory and Critical Medicine, The Second Affiliated Hospital of Shanxi Medical University, Taiyuan, Shanxi 030001, China; ^3^ Department of Respiratory and Critical Medicine, Shanxi Provincial People’s Hospital Affiliated to Shanxi Medical University, Taiyuan, Shanxi 030012, China; ^4^ Division of Allergy and Immunology, Department of Medicine, Beth Israel Deaconess Medical Center, Harvard Medical School, Boston, MA 02215, USA

**Keywords:** long non-coding RNA-LET, NSCLC, prognosis, Notch signaling

## Abstract

Long non-coding RNAs (lncRNAs) have been found to be dysregulated in a variety of tumors. The lncRNA-Low Expression in Tumor (LET) is a recently identified lncRNA, but its expression pattern and biological significance in human non-small cell lung cancer (NSCLC) are still largely unknown. In this study, we found that lncRNA-LET was significantly downregulated in human NSCLC lung tissues and cell lines. Decreased lncRNA-LET expression was strongly associated with advanced tumor stages and poorer overall survival of NSCLC patients. Functionally, overexpression of lncRNA-LET in NSCLC H292 cells significantly suppressed cell proliferation, migration and invasion, and promoted cell cycle arrest and apoptosis, while knockdown of lncRNA-LET in NSCLC H1975 cells showed an opposite effect, pointing to a tumor-suppressive role for lncRNA-LET in NSCLC. Mechanistically, we demonstrated that lncRNA-LET overexpression significantly reduced the expression of Notch1 intracellular Domain (NICD1) in H292 cells while knockdown of lncRNA-LET increased NICD1 expression in H1975 cells. Similarly, NSCLC lung tissues with high levels of lncRNA-LET had lower NICD1 expression. Thus, our results provide a strong rationale for lncRNA-LET to be used as a prognostic indicator and a potent therapeutic target for NSCLC patients, and highlight a novel lncRNA-LET/Notch axis in regulating NSCLC cell fate and tumor progression.

## INTRODUCTION

Lung cancer is the most common cancer and the leading cause of cancer deaths. The non-small cell lung cancers (NSCLC) account for approximately 85% of all lung cancer cases, which are at locally advanced or metastatic stage at diagnosis [1, 2]. Although NSCLC has a large worldwide prevalence with a high mortality rate, there remains a lack of specific and sensitive tools for early diagnosis and targeted therapies. Therefore, it is of paramount importance to understand the pathophysiological mechanisms contributing to NSCLC for developing new treatment strategies and improving the overall prognosis of NSCLC patients.

Microarrays and high-throughput sequencing have revolutionized our ability to uncover the widespread expression of non-coding RNAs, including microRNAs (miRNA) and long non-coding RNAs (lncRNAs), which impact biologic responses through the regulation of mRNA transcription and/or translation [3, 4]. lncRNAs are largely polyadenylated and more than 200 nucleotides in length transcripts, involved in gene expression through epigenetic and transcriptional regulation, splicing, imprinting and subcellular transport. lncRNA dysregulation has increasingly been recognized to contribute to the development and progression of some human malignancies including lung cancer [5-8]. In NSCLC patients, some of these lncRNAs are associated with different TNM stages or specifically overexpressed in one of the lung cancer subtypes while others are involved in drug resistance [8-10]. Those findings suggest the important roles of lncRNAs in the pathogenesis of NSCLC. However, only a small number of lncRNAs have been well characterized, whereas functions of most lncRNAs remain to be elucidated [11].

LncRNA-Low Expression in Tumor (lncRNA-LET, NCBI number AK055007), a recently identified lncRNA located at chromosome 15q24.1, has been demonstrated to be downregulated in several types of cancer, including hepatocellular carcinomas [12], esophageal squamous cell carcinoma [13] and gallbladder cancer [14]. However, the expression and function of lncRNA-LET in NSCLC are still largely unknown. In this study, we found that lncRNA-LET was significantly downregulated in human NSCLC lung tissues and cell lines, and low lncRNA-LET expression predicted shorter survival of NSCLC patients. Mechanistically, lncRNA-LET suppressed NSCLC cell proliferation and migration, and promoted cell apoptosis at least in part by its ability to downregulate Notch signaling, which is predominately activated and involved in NSCLC development and progression.

## RESULTS

### lncRNA-LET expression is significantly decreased in NSCLC tissues and cell lines

**Figure 1:**
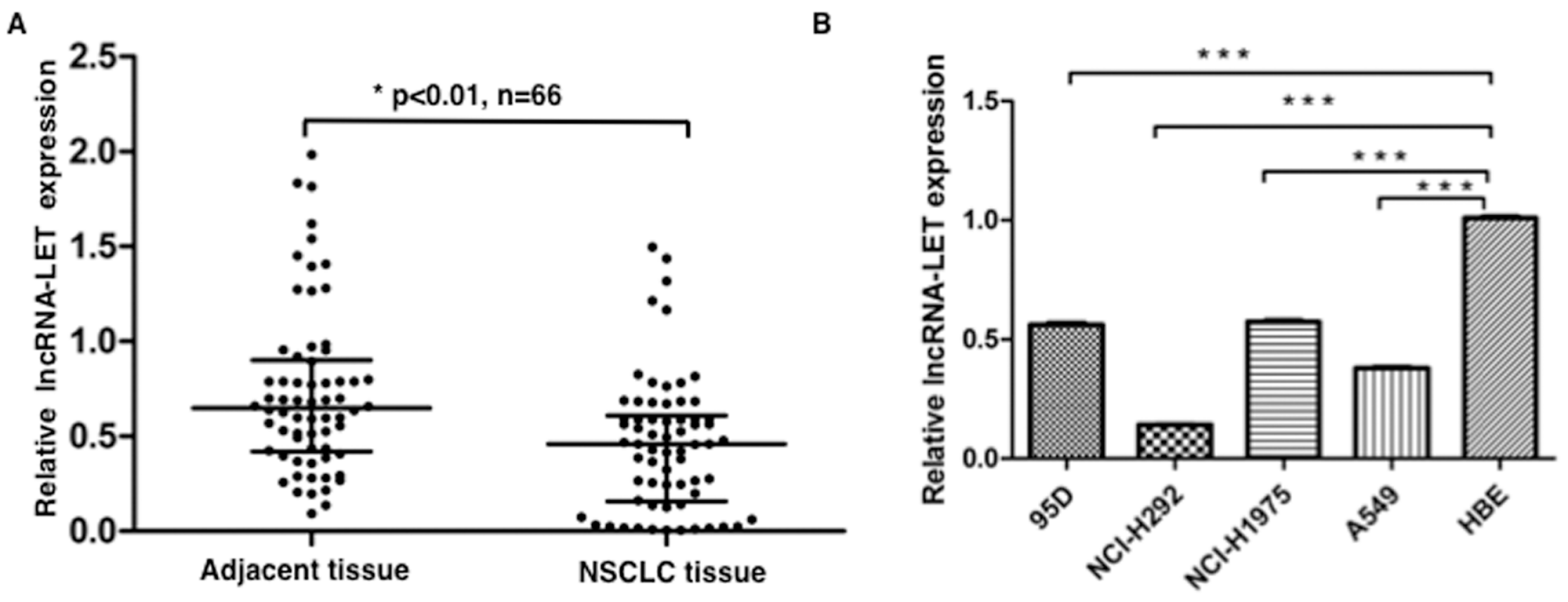
lncRNA-LET expression is downregulated in NSCLC tissues and cell lines **(A)** The expression levels of lncRNA-LET were measured by qRT-PCR in NSCLC tissues and adjacent normal lung tissues (n=66), and **(B)** in four NSCLC cell lines and normal human epithelial cell (HBE). GAPDH gene expression was used as normalization control. Data are shown as mean ± SD from three independent experiments. ^***^P<0.001.

We measured the expression levels of lncRNA-LET in 66 NSCLC tissue samples and paired adjacent non-cancerous tissues by quantitative RT-PCR (qRT-PCR), and found that lncRNA-LET expression was significantly downregulated in the NSCLC tissues compared with adjacent normal tissues (Figure 1A). Also, we assessed the expression levels of lncRNA-LET in four human NSCLC cell lines (A549, 95D, NCI-H292, and NCI-H1975) and one normal human bronchial epithelial (HBE) cell line. As shown in Figure 1B, significantly decreased lncRNA-LET expression was also found in all four human NSCLC cell lines compared with normal HBE cell line. These data indicated that lncRNA-LET expression was inhibited in NSCLC carcinogenesis.

### Low lncRNA-LET expression is associated with poor prognosis of NSCLC patients

**Figure 2:**
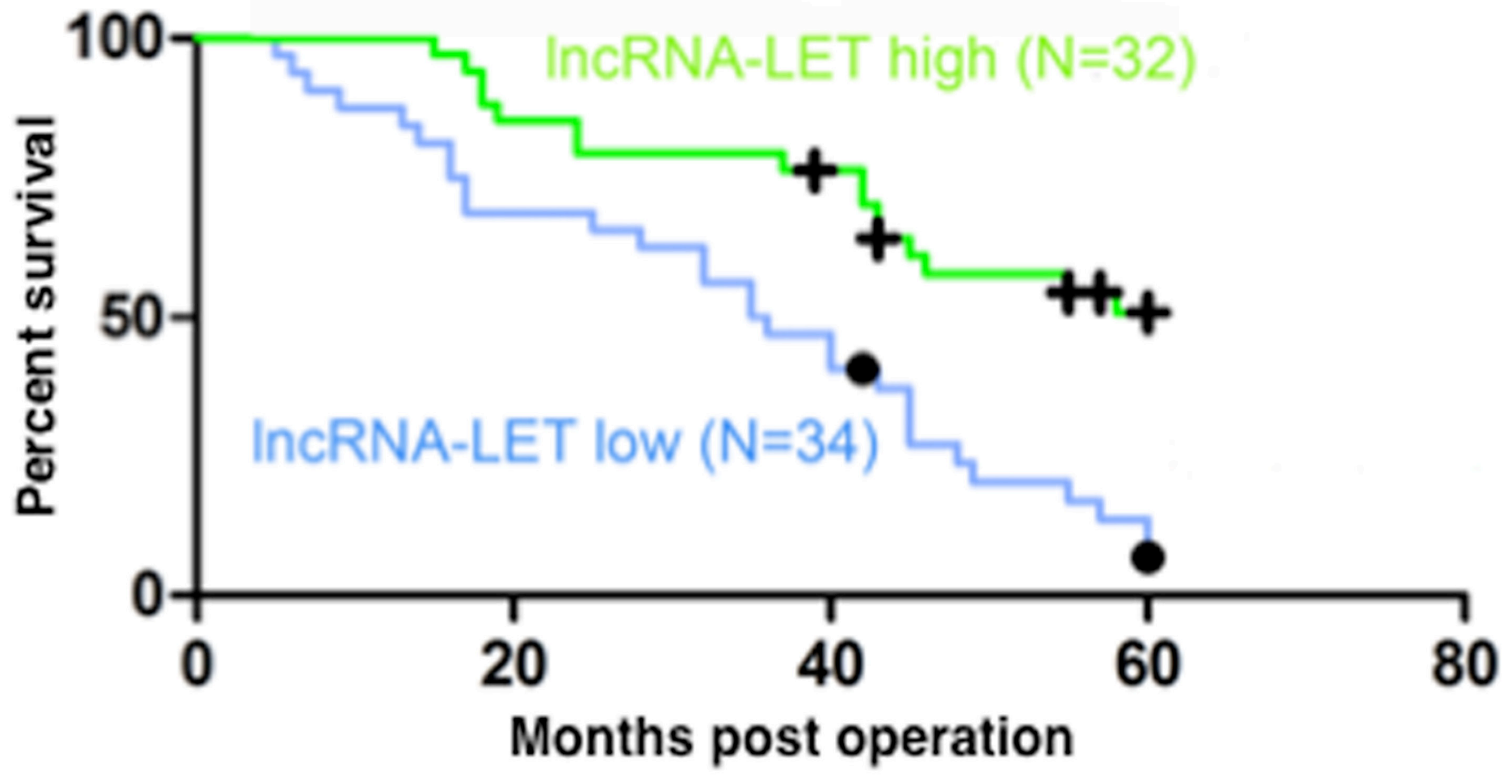
Low lncRNA-LET expression predicts poor prognosis of NSCLC patients Kaplan-Meier analysis of overall survival (log-rank test) in 66 NSCLC patients who were divided into a high lncRNA-LET expression group (green line, n=32) and a low LET expression group (blue line, n=34) using the median of the lncRNA-LET expression levels as the cut-off value.

To evaluate the correlation between the expression levels of lncRNA-LET and the prognosis of patients with NSCLC, the 66 NSCLC patients were divided into a high lncRNA-LET expression group (n=32) and a low lncRNA-LET expression group (n=34) using the median as the cut-off value based on the lncRNA-LET expression levels obtained by qRT-PCR. The Kaplan–Meier survival analysis and log-rank tests showed that the 5-year overall survival rate was less than 15% in the low lncRNA-LET expression group but increased to more than 50% in the high lncRNA-LET expression group (Figure 2), indicating that decreased lncRNA-LET expression was associated with negative prognosis of NSCLC patients. To confirm this finding, we further analyzed the correlation between lncRNA-LET expression levels and clinicopathological features in these NSCLC patients. As shown in Table 1, patients with low lncRNA-LET expression had poorer differentiation degree, greater lymph node metastasis and higher TNM stage than those with high lncRNA-LET expression, but there was no significant correlation between the expression levels of lncRNA-LET and gender or age. Multivariate analysis demonstrated that a low expression level of lncRNA-LET was a significant predictor of subsequent metastasis (Table 2). Overall, these data suggest that Low lncRNA-LET expression was associated with the clinical progression of human NSCLC, and lncRNA-LET might serve as a predictive biomarker for NSCLC patients.

**Table 1:** Correlation between lncRNA-LET expression and clinicopathologic characteristics in NSCLC patients

Characteristics	n	lncRNA-LET expression		*χ*^2^-Value	*P*-Value
		Low	High		
Gender					
Male	46	22	24	0.026	0.871
Female	20	10	10		
Age					
<55	18	8	10	2.802	0.246
55-64	35	15	20		
≥65	13	9	4		
T status					
T1-2	50	20	30	9.504	0.009
T3-4	16	12	4		
N status					
N0	32	14	18	6.445	0.040
N1/2	34	18	16		
Clinical stage					
I–II	32	11	21	4.951	0.026
III–IV	34	21	13		
Histological grade					
Well and moderately	20	6	14	3.926	0.048
Poorly and others	46	26	20		
Lymphatic metastasis					
Negative	21	3	18	14.422	<0.001
Positive	45	29	16		

**Table 2:** Multivariate analysis of risk factors for metastasis as the first recurrence event in NSCLC patients (Cox proportional hazards regression model)

Risk factors	*b*	SE (*b*)	Wald chi-square	*P*	RR	HR 95% CI	
						low limit	High limit
Gender	0.604	0.411	2.167	0.141	1.830	0.818	4.092
Age	0.253	0.255	0.986	0.321	1.288	0.782	2.121
Pathological type	-0.595	0.339	3.067	0.080	0.552	0.284	1.073
T Status	-0.227	0.249	0.832	0.362	0.797	0.489	1.298
N status	0.584	0.231	6.395	0.011	1.792	1.140	2.817
Clinical stage	0.855	0.405	4.462	0.035	2.352	1.064	5.201
Histological grade	0.530	0.270	3.854	0.050	1.700	1.001	2.886
lncRNA-LET expression	-1.027	0.482	4.547	0.033	0.358	0.139	0.920

Note: HR, hazard ratio; CI, confidence interval.

### lncRNA-LET inhibits migration and invasion of NSCLC H292 cells

As clinical association analysis showed that lncRNA-LET downregulation was positively correlated with more aggressive phenotype of NSCLC, we speculated that lncRNA-LET might modulate cancer cell migration and invasion. To verify this hypothesis, NSCLC cell line NCI-H292, expressing the lowest level of lncRNA-LET (Figure 1B), was selected for gain-of-function experiments by being infected with lentivirus containing lncRNA-LET (p-Lenti-IncRNA-LET) or empty vectors (control). Following transfection, lncRNA-LET expression was significantly increased compared to the cells transfected with empty vector (data not shown).

**Figure 3:**
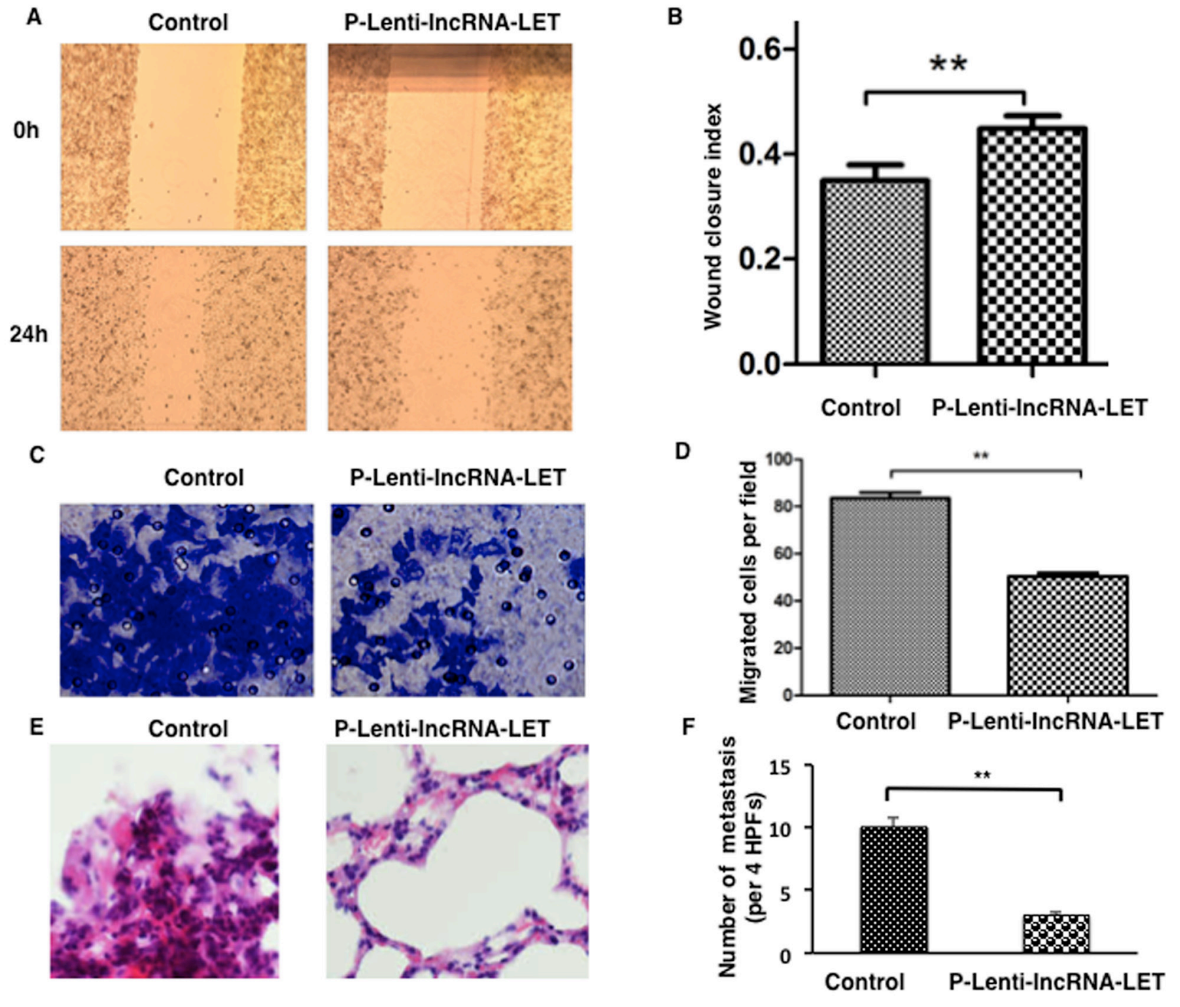
Overexpression of lncRNA-LET inhibits migration and invasion of NSCLC H292 cells both *in vitro* and *in vivo* NSCLC H292 cells infected with lentivirus expressing lncRNA-LET (P-Lenti-IncRNA-LET) or empty vectors (control) were used in the experiments. Representative images are presented on the left and the histograms for statistical analyses on the right. **(A)**, **(B)** Wound healing assays were used to assess the migration ability of the cells. **(C)**, **(D)** Transwell assays using the Matrigel invasion chamber were employed to examine invasion capacities of the cells. **(E)**, **(F)** Histological analysis of the numbers of lung metastases in nude mice that received intravenous tail injections of control or lncRNA-LET overexpressing H292 cells. E. H&E stained sections of lung tissues with metastasis. F. The numbers of metastases were counted and analyzed. Data are presented as the mean ± SD of 6 mice per group. HPF, high power field. ^**^ P<0.01.

Wound healing assays revealed that lncRNA-LET overexpression prohibited migration of NCI-H292 (Figure 3A and 3B). lncRNA-LET overexpression also significantly suppressed the invasive capacities of NCI-H292 as determined by transwell experiments (Figure 3C and 3D).

To investigate the effects of lncRNA-LET overexpression on NSCLC cell invasion and metastasis *in vivo*, NCI-H292 cells infected with lentivirus containing lncRNA-LET or control vectors were injected into the tail vein of each anesthetized nude mouse (n=6 per group). We compared the rates of lung colonization four weeks after cell injections. The tail vein injection of lncRNA-LET-overexpressing H292 cells resulted in the engraftment of fewer cells in the lung compared with the control cells, as examined by lung histology (Figure 3E and 3F). Thus, the both *in vitro* and *in vivo* results indicated that lncRNA-LET overexpression inhibited NSCLC metastasis by regulating cell migration and invasion.

### lncRNA-LET overexpression leads to apoptosis of NSCLC H292 cells

**Figure 4:**
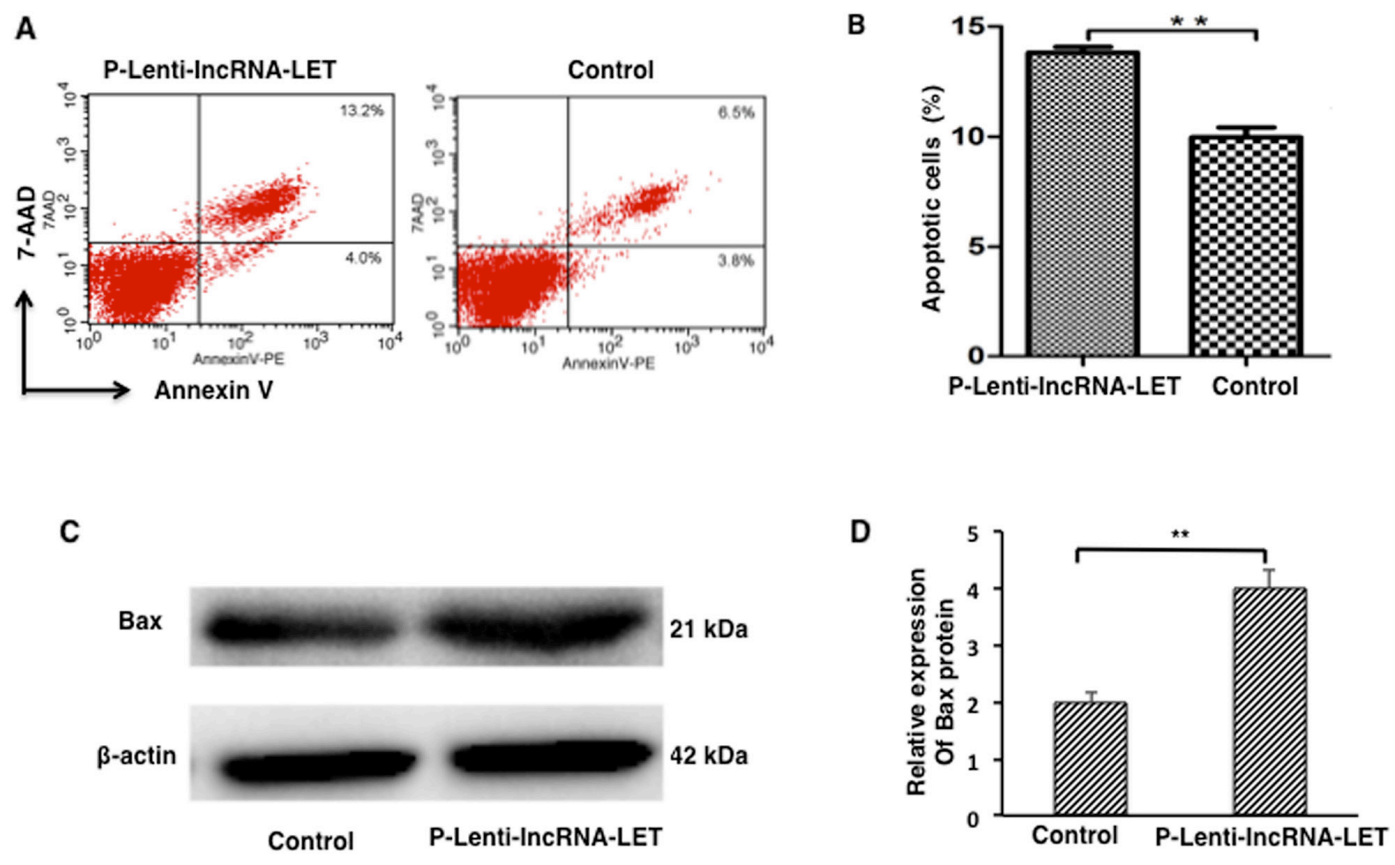
lncRNA-LET overexpression leads to apoptosis of NSCLC H292 cells NSCLC H292 cells infected with lentivirus expressing lncRNA-LET (P-Lenti-IncRNA-LET) or empty vectors (control) were used in the experiments. **(A)** Representative dot blots of flow cytometry to assess cell apoptosis after Annexin V/7-AAD staining. **(B)** Apoptotic cell percentages of total cells by flow cytometry. **(C)** Expression of apoptotic factor Bax protein by Western blotting. **(D)** Bax quantitation obtained from densitometry analysis of the blots after normalization to β-actin. Data represent the mean ± S.D. from three independent experiments. ^**^P<0.01.

Cell proliferation, metastasis and apoptosis are essential cancer cell functions. Next, we assessed the effect of lncRNA-LET on cell apoptosis of NSCLC H292 cells. The results demonstrated that lncRNA-LET overexpression significantly promoted apoptosis in NSCLC H292 cells (Figure 4A and 4B). Western blotting analysis revealed that expression of the pro-apoptotic factor Bax was greatly increased in lncRNA-LET overexpressing H292 cells (Figure 4C and 4D) compared with the control cells.

### lncRNA-LET suppresses NSCLC H292 cell proliferation by inducing cell cycle arrest

**Figure 5:**
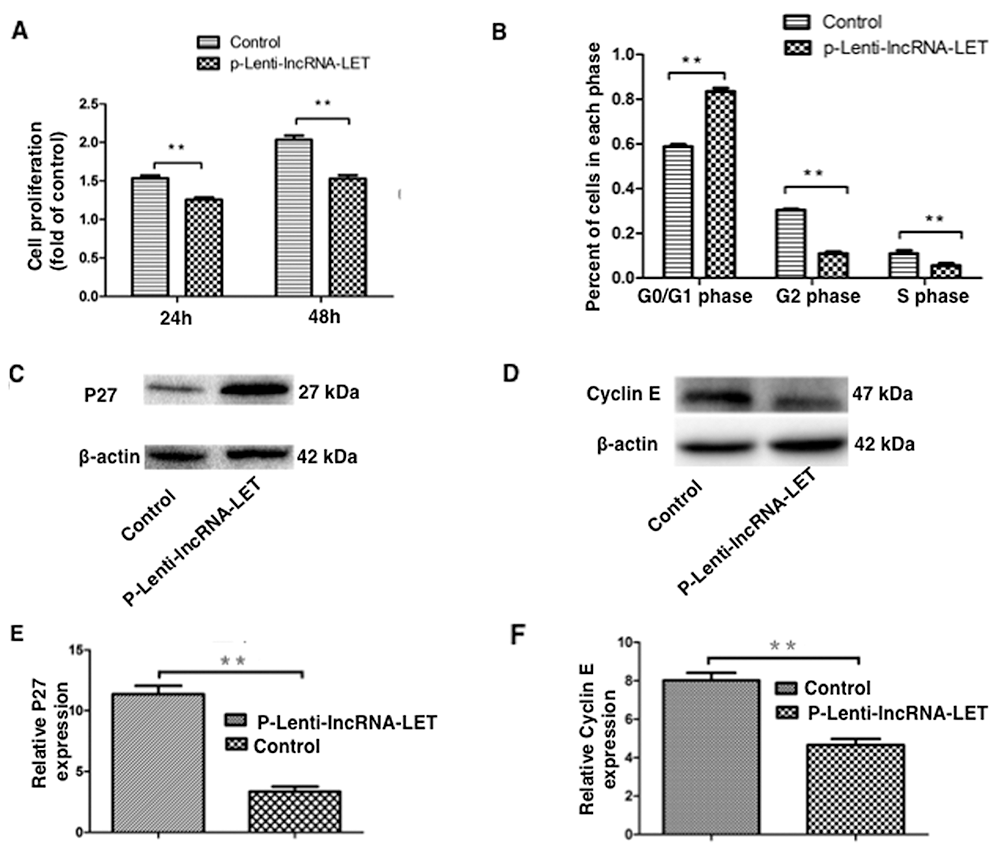
lncRNA-LET overexpression suppresses NSCLC H292 cell proliferation by inducing cell cycle arrest NSCLC H292 cells infected with lentivirus expressing lncRNA-LET (P-Lenti-IncRNA-LET) or empty vectors (control) were used in the experiments. **(A)** H292 cell proliferation was measured by CCK-8 assays at indicated times. Data are presented as the mean ± SD of three independent experiments. ^**^P<0.01. **(B)** The percentage of cells in each of cell-cycle phases was determined by flow cytometry. **(C)**, **(E)** Expression of the G0/G1 arrest marker P27 and **(D)**, **(F)** G1/S transition marker Cyclin E were measured by western blotting and densitometry analysis. Data represent the mean ± S.D. from three independent experiments (E, F). ^**^P<0.01.

We then examined the effect of lncRNA-LET expression on the proliferation of H292 cells. Compared to empty vector- infected cells (control), lncRNA-LET overexpressing H292 cells showed significantly decreased proliferation 24h or 48h after incubation, as determined by CCK8 assay (Figure 5A). These findings indicated that lncRNA-LET might function to suppress the proliferation of NSCLC cells.

As dysregulation of cell cycle transition is a hallmark of cancer cells [15], we further investigated whether the effect of lncRNA-LET on NSCLC cell proliferation was due to altered cell cycle progression. As demonstrated in Figure 5B, lncRNA-LET overexpression caused a dramatic decrease in S-phase and accumulation in G0/G1-phase of H292 cells. Western blotting showed that the G0/G1 arrest marker p27 expression was greatly increased (Figure 5C), whereas G1/S transition marker cyclin E expression was greatly decreased in lncRNA-LET overexpressing H292 cells (Figure 5D).

**Figure 6:**
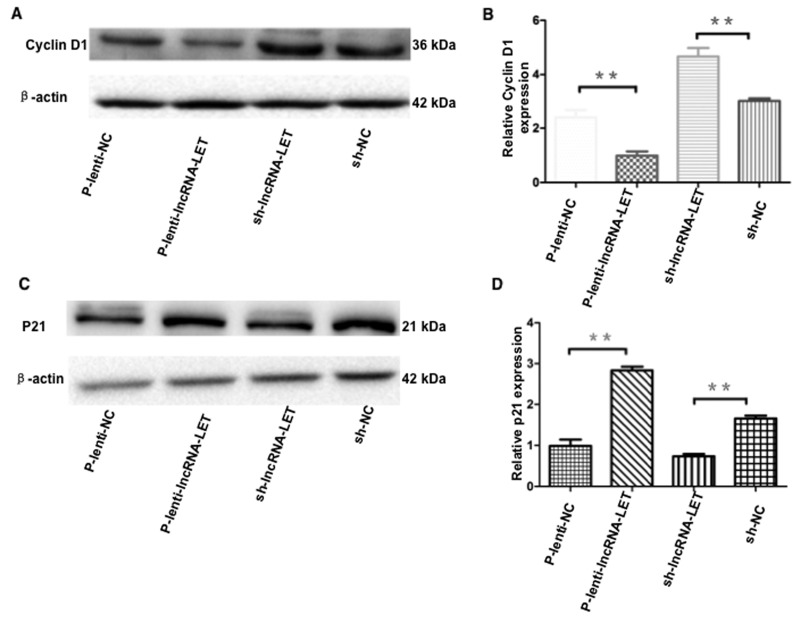
Effect of overexpression or knockdown of lncRNA-LET on expression of cyclin D1 and p21 in NSCLC cells NSCLC H292 cells were infected with lentivirus expressing lncRNA-LET (P-Lenti-IncRNA-LET) or empty vectors (p-lenti-NC) for gain-of-function experiments. For loss-of-function experiments, H1975 cells were used after transfection with shRNA targeting lncRNA-LET (sh-lncRNA-LET) or control shRNA (sh-NC). Expression of the G1/S checkpoint effector molecule cyclin D1 **(A, B)** and p21 **(C, D)** were measured by western blotting and densitometry analysis. Data represent the mean ± S.D. from three independent experiments (B, D). ^**^P<0.01.

The cell cycle is tightly regulated by a variety of proteins. We further examined expression levels of the cell cycle G1/S checkpoint key effector molecule cyclin D1 and p21. Western blotting data showed that overexpression of lncRNA-LET significantly decreased cyclin D1 and increased p21 expression in H292 cells (Figure 6). To ensure the results obtained from using only one NSCLC cell line and gain-of-function experiments were not due to cell type-specific or artificial expression effect, we employed a second NSCLC cell line - H1975 cells, transfected with shRNA targeting lncRNA-LET, and performed loss-of-function experiments. Knockdown of lncRNA-LET significantly increased cyclin D1 and decreased p21 expression in H1975 cells, showing an opposite effect compared to lncRNA-LET overexpressing H292 cells (Figure 6).

### lncRNA-LET reduces Notch1 (NICD1) expression in NSCLC cell lines and tissues

**Figure 7:**
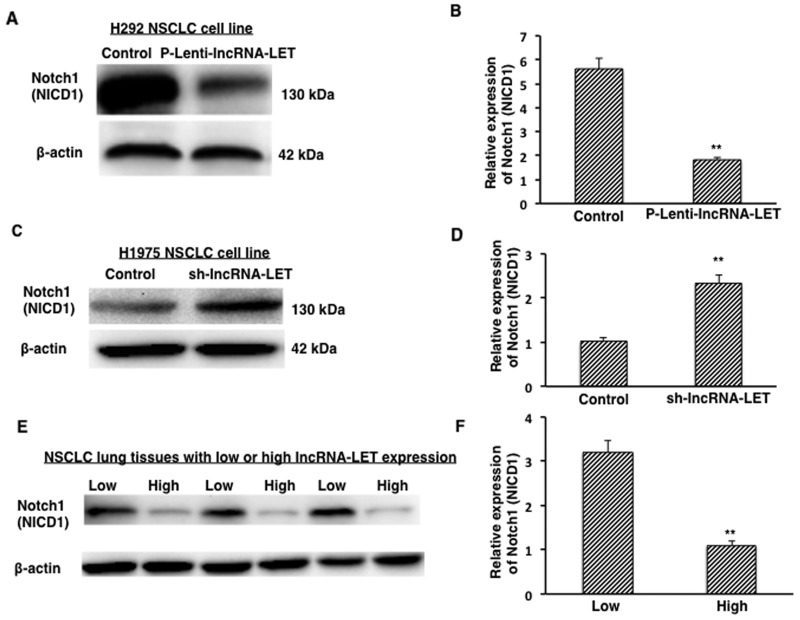
Expression of active form of Notch1 (NICD1) in NSCLC tissues and cells **(A)**, **(B)** Overexpression of lncRNA-LET (P-Lenti-IncRNA-LET) reduced NICD1 expression in H292 cells. **(C)**, **(D)** Knockdown of lncRNA-LET (sh-IncRNA-LET) increased NICD1 expression in H1975 cells. **(E)**, **(F)** Expression of NICD1 protein from NSCLC tissues with low (n=3) or high (n=3) LncRNA-LET expression levels. Relative NICD1 expressions from densitometry analysis of the blots after normalization to β-actin represent the mean ± S.D. from three independent experiments (B, D), and from ten NSCLC lung tissue samples (F). ^**^P<0.01.

In NSCLC cell lines, it has been reported that the expression of the active form of Notch1 (Notch1 intracellular domain, NICD1) leads to increased proliferation activity, malignant transformation, and tumor growth [16]. To investigate the potential mechanism of lncRNA-LET in regulating NSCLC cell fate, we measured the expression of Notch 1 intracellular domain (NICD1) in NSCLC cells. We found that overexpression of lncRNA-LET significantly decreased expression of the NICD1 in H292 cells (Figure 7A and 7B) while knockdown of lncRNA-LET significantly increased NICD1expression in H1975 cells (Figure 7C and 7D). These findings by lncRNA-LET overexpression and knockdown experiments were further confirmed in the lung tissues from NSCLC patients. As shown in Figure 7E and 7F, NSCLC lung tissues with low lncRNA-LET levels had stronger Notch1 (NICD1) expression than those with high lncRNA-LET levels. Taken together, our results from both NSCLC cells and tissues suggest a novel lncRNA-LET/Notch axis in regulating NSCLC cell fate and tumor progression.

## DISCUSSION

In this study, we examined lncRNA-LET expression levels in lung tissue samples from 66 patients with NSCLC, and found that lncRNA-LET expression is significantly downregulated in NSCLC tissues compared with adjacent normal lung tissues. Patients with lower expression levels of lncRNA-LET in NSCLC tissues show greater lymph node metastasis, higher TNM stage and poorer overall survival, suggesting that lncRNA-LET may function as a tumor suppressive gene in NSCLC development and progression. This tumor suppressive function of lncRNA-LET was further investigated by using NSCLC cell lines with overexpression or knockdown of lncRNA-LET. The results have demonstrated that overexpression of lncRNA-LET in NSCLC H292 cells decreases cell growth, migration and invasion, and increases apoptosis, while knockdown of lncRNA-LET shows an opposite effect in H1975 cells. Mechanistic study has revealed that NSCLC lung tissues with low lncRNA-LET levels have stronger activated Notch1 (NICD1) expression than those with high lncRNA-LET levels, and that lncRNA-LET inhibits the Notch signaling pathway by targeting Notch1 in NSCLC cells. Thus, Our results highlight a novel lncRNA-LET/Notch axis in regulating NSCLC cell fate and tumor progression.

We measured expression levels of lncRNA-LET in four NSCLC cell lines (A549, 95D, NCI-H292 and NCI-H1975) and a normal human bronchial epithelial (HBE) cell line. Quantitative RT-PCR data showed that all four human NSCLC cell lines expressed lower levels of lncRNA-LET compared with normal HBE cell line. Notably, these results are partially contradictory to those of Liu et al [17], who reported that expression level of lncRNA-LET in HBE cells was not necessarily higher than those in NSCLC cell lines, among which A549 cells presented the highest level. The contradictory findings could be due to different sources of cell lines and/or technical reasons. For example, for culture of HBE cells they used RPMI1640 while we used α-MEM medium.

In order to explore the role of lncRNA-LET on NSCLC cell biological function, NSCLC cell line NCI-H292, having the lowest expression of lncRNA-LET among four NSCLC cell lines, was selected and infected by lentivirus containing the lncRNA-LET expression vector. Our results demonstrated that overexpression of lncRNA-LET inhibited proliferation and migration of NSCLC H292 cells, and led to the induction of cell cycle arrest and cell apoptosis. We next investigated the possible mechanisms underlying cell cycle arrest by lncRNA-LET. Western blotting data showed that overexpression of lncRNA-LET significantly decreased cyclin E and D1 expression, and increased p27 and p21 expression in H292 cells. In contrast, knockdown of lncRNA-LET significantly increased cyclin D1 expression and decreased p21 expression in H1975 cells. Progression through the cell cycle is governed by a family of cyclin-dependent kinases, which are activated by binding cyclins including cyclin E and D, and inhibited by the kinase inhibitor protein family including p21 and p27 [18]. The p27 is a negative regulator of the protein kinase CDK2/cyclin E and can block the cell cycle at G0/G1 phase [19-21], while cyclin D1 and p21 are key effector molecules regulating G1 into S phase [22, 23]. Furthermore, cyclin D1 associates with p21 in metastatic breast cancer cells [24], and is a well-characterized oncogene that is frequently overexpressed in human lung carcinomas [25]. Thus, our findings suggest that regulation of p27/cyclin E and p21/cyclin D1 pathways may be one of the mechanisms by which lncRNA-LET blocks cell cycle progression and inhibits cell proliferation in NSCLC cells. Yet, the exact molecular mechanisms behind their interaction need further investigation.

The Notch signaling pathway is deregulated in numerous solid tumors including NSCLC [26-28]. It has been documented that the expression of Notch1 in NSCLC tissues is significantly higher compared with the normal lung tissues [29-32]. The expression of activated Notch1 (NICD1) in the pulmonary epithelium of mice can induce lung adenomas [33]. After Notch1 ablation *in vivo*, there is a dramatic decrease in tumor initiation and burden in a mouse model of lung adenocarcinoma, demonstrating that Notch1 is implicated in the initiation, proliferation and survival of NSCLC models in preclinical studies [16]. A recent meta-analysis indicates that Notch signaling is a valuable biomarker for predicting the progression of NSCLC, and that the higher expression of Notch signaling is associated with a greater possibility of lymph node metastasis, higher TNM stages and poor survival of NSCLC patients [34]. Although Notch has been confirmed as a key player in the pathogenesis of lung cancer, little is known about how Notch pathway is regulated locally in the lung. In this study, we demonstrated that NSCLC lung tissues with low lncRNA-LET levels showed stronger Notch1 (NICD1) expression than those with high lncRNA-LET levels. Thus, our results provide new insights towards understanding the regulation of Notch signaling in NSCLC development and progression.

Recent evidences indicate that there is a crosstalk between Notch and non-coding RNA, mainly miRNAs [35, 36]. Notch can regulate expression of a number of miRNAs; at the same time, Notch ligands or Notch receptors are regulated by miRNAs [35]. Induction of miR-34a decreased the expression of Notch1 and its downstream targets including Cyclin D1 and Bcl-2, impairing Notch signaling, cell proliferation, and invasion and inducing apoptosis in NSCLC cells [37]. In this study, we investigated whether lncRNAs, in addition to miRNAs, could regulate expression of Notch1 in NSCLC cells. Our results showed that overexpression of lncRNA-LET significantly decreased expression of the active form of Notch1 (NICD1) in NSCLC H292 cells while knockdown of lncRNA-LET significantly increased NICD1expression in H1975 cells. These results suggest that lncRNA-LET -triggered tumor-suppressive activity could be due to its ability to regulate Notch signaling pathway. However, the detailed mechanisms of how lncRNA-LET regulates Notch pathway remain unclear and will be the focus of prospective studies. It has been reported that lncRNA-LET can inhibit cancer cell growth and migration through regulating MAPK/ERK pathway [38], Wnt/β-catenin pathway [17] or p53 expression [39]. Notably, Notch pathway is known to interplay with signaling pathways mentioned above [16, 40, 41]. Therefore, the downregulation of NICD1 by lncRNA-LET might not be a direct effect, which needs to be further examined in our future work. Nevertheless, the results from NSCLC patients’ samples and cell lines have demonstrated a novel link connecting lncRNA-LET with Notch pathway.

In summary, we have demonstrated that low levels of lncRNA-LET expression are strongly associated with poor survival in NSCLC patients. lncRNA-LET inhibits NSCLC cell proliferation and migration, and promotes cell apoptosis at least in part via downregulation of the activated Notch1, which provides new insights towards understanding the regulation of Notch signaling pathway in NSCLC progression. These results may open new prospects for targeting lncRNA-LET/Notch axis in the treatment of NSCLC patients.

## MATERIALS AND METHODS

### Patient tissue samples

Sixty-six NSCLC tissue samples and adjacent non-cancerous lung tissues (collected postoperatively from January 2010 to December 2010) were obtained from Shanxi Provincial People’s Hospital Affiliated to Shanxi Medical University (Shanxi, China). Upon surgical removal of specimens, each sample was snap-frozen in liquid nitrogen and stored at-80°C prior to RNA isolation and qRT-PCR analysis. Patients recruited to this study had not received any preoperative treatments. Patients with NSCLC were staged according to the seventh edition of the tumor node metastasis (TNM) staging system of the International Union Against Cancer (UICC). The data do not contain any information that could identify the patients, and all samples were obtained with informed consent. All patients were regularly followed up with an observation period of 60 months. Seven patients dropped out at the later stage of follow-up. This study was approved by Shanxi Provincial People’s Hospital Affiliated to Shanxi Medical University (Shanxi, China). Human subjects research was performed in accordance with the Declaration of Helsinki.

### Cell culture

Four human NSCLC cell lines (A549, 95D, NCI-H292 and NCI-H1975) used in this study were purchased from the Cell Bank of the Chinese Academy of Science (Shanghai, China). The normal human bronchial epithelial (HBE) cells were a generous gift from the College of Occupational and Environmental Health of Shanxi Medical University, China. All four NSCLC cell lines were cultured in RPMI 1640 (HyClone), and HBE cells were cultured in α-MEM containing 10% fetal bovine serum (FBS Gibco), 100 U/ml penicillin, and 100 mg/ml streptomycin (Sigma). Cells were maintained in a humidified incubator at 37°C in the presence of 5% CO_2_. All cell lines were passaged for fewer than 3 months.

### RNA preparation and quantitative RT-PCR

Total RNA from tissues and cells was extracted using a MiniBEST Universal RNA Extraction Kit (TaKaRa, Dalian, China). RNA was reverse-transcribed into cDNA using a Primer-Script™ RT Master Mix kit (TaKaRa, Dalian, China). The cDNA template was amplified by real-time PCR using a SYBR Premix Ex Taq™ IIkit (TaKaRa, Dalian, China). Glyceraldehyde-3-phosphatedehydrogenase (GAPDH) was used as an internal control, and lncRNA-LET values were normalized to GAPDH. RT-PCR reactions were performed using the CFX96 Touch system (Bio-Rad, USA). The relative expression of mRNAs was calculated using the 2^-∆∆Ct^ methods. The primer sequences were as follows: GADPH: 5’GTCAACGGATTTGGTCTGTATT-3’ (forward), 5’-AGTCTTCTGGGTGGCAGTGAT-3’ (reverse); lncRNA-LET: 5’-CCTTCCTGACAGCCAGTGTG-3’ (forward), 5’-CAGAATGGAAATACTGGAGCAAG-3’ (reverse).

### Lentivirus infection

For overexpression studies, a lentivirus vector containing lncRNA-LET (pLV-CMV-lnRNALET-hEF1a-EGFP-2A-Puro) and a control lentivirus vector without the lncRNA-LET gene (pLV-hEF1a-EGFP-2A-Puro) were constructed (SyngenTech, Beijing, China). Purified lncRNA-LET recombinant lentivirus particles (1×10^8^ TU/ml) and control lentivirus particles (1×10^8^ TU/ml) were added to NCI-H292 cells at MOI 50. Forty-eight hours after infection, puromycin (1 μg/ml) was used to screen for puromycin-resistant infected cells. Stably infected cells were harvested and passaged continuously for further research.

For knockdown studies, the shRNAs targeting lncRNA-LET were designed. The nucleotide sequences of shRNA for lncRNA-LET were as the followings:

lncRNA-LET shRNA-1 (shLET 1): GCAAATGAATCTCTAGTTTCCCGAAGGAAACTAGAGATTCATTTGC;

lncRNA-LET siRNA-2 (shLET-2): GGAGTAAAGGGAAAGAGTTGCCGAAGCAACTCTTTCCCTTTACTCC;

lncRNA-LET shRNA-3 (shRNA-3): GCATGTGGTAGGTTAGATTTGCGAACAAATCTAACCTACCACATGC. Negative control shRNA (shLET-NC) were purchased from SyngenTech (Beijing, China). Purified shRNA for lncRNA-LET recombinant lentivirus particles and control lentivirus particles were added to NCI-H1975 cells at MOI 80. Forty-eight hours after infection, puromycin (0.2 μg/ml) was used to screen for puromycin-resistant cells. Stably infected cells were harvested and passaged continuously. Quantitative detection of silencing efficiency by qRT-PCR showed that shRNA-2 had the highest efficiency of silence (42%). So NCI-H1975 infected with shRNA-2 and control shRNA were selected for further research.

### Cell migration and invasion assays, and the *in vivo* metastasis model

For the migration assays, 5×10^5^ cells, infected with lncRNA-LET or control lentiviruses, in serum-free RPMI1640 media were seeded in six-well plates and cultured in RPMI-1640 medium. After 48 h, cell layers were wounded using the tip of 200 μl pipette. After washing cells three times with PBS, the serum-free RPIM-1640 medium was added to the plates and incubated within a humidified atmosphere containing 5% CO2 at 37°C for 48 h. Wound closure was observed under a light microscope and measured using AxioVision version 4.7 software (Carl Zeiss Meditec, Dublin, CA, USA).

For the invasion assays, cells in serum-free RPMI1640 media were placed into the upper chamber of an insert (8.0mm, Millipore, Boston, MA), which was pre-coated with Matrigel. The chambers were then incubated for 24h in culture medium with 20% FBS in the bottom chambers before examination. The cells on the upper surface were scraped and washed away, whereas the migrated cells on the lower surface were fixed with 95% ethanol for 30 min and stained with 0.05% crystalviolet for 30 min. Finally, invasive cells were counted under a microscope and the relative number was calculated. Experiments were performed in triplicate.

For lung metastasis model, 2×10^6^ H292 cells infected with lentivirus expressing lncRNA-LET or empty vector, were injected into the tail veins of nude mice. Four weeks later, we quantified metastatic nodules in the lung by histology examination of H&E stained lung sections. The animal study was approved by the Institutional Animal Care and Use Committee of the Shanxi Provincial People’s Hospital Affiliated to Shanxi Medical University (Shanxi, China).

### Cell proliferation

NCI-H292 cells infected with lncRNA-LET expressing lentivirus or control lentivirus were trypsinized and resuspended, and then 5000 cells per well were seeded into 96-well plates. Cell proliferation was evaluated with a Cell Counting Kit-8 (Dojindo, Beijing, China) according to the manufacturer’s protocol. Experiments were performed in triplicate.

### Flow cytometric analysis

Cells infected with lncRNA-LET expressing lentivirus or control lentivirus were plated in 6-well plates. After 48 h incubation, the cultures were incubated with propidium iodide for 30 min in the dark. Cultures were then collected and analyzed for cell cycle progression using a flow cytometer (Beckman, USA). The cultures were also stained with annexin V/7-AAD for cell apoptosis.

### Western blot analysis

NSCLC lung tissues or cells were lysed on ice in RIPA lysis buffer (Beyotime, Shanghai) supplemented with protease inhibitors (Roche). The lysates were then collected and centrifuged at 14,000 rpm for 12 min. The supernatants were collected, and protein content was determined by Bradford assay. Total proteins were resolved using 12% SDS-PAGE separating gel (CWBiotech, Beijing, China) and blotted onto a PVDF membrane (Millipore, USA). Membranes were then blocked with 5% non-fat powdered milk intriethanolamine-buffered saline solution with Tween (TBS-T) at room temperature for 2 h, then incubated overnight with primary antibody at 4°C. Anti-p27(1:1000), Anti-p21(1:1000), anti-Bax (1:1000), anti-Cyclin D1 (1:1000), anti-Cyclin E (1:1000), anti-beta-actin (1:2000) or anti-Notch1 intracellular domain (NICD1) (1:2000). All primary antibodies were purchased from Abcam, USA. After three washes in TBS-T for 5 min, the membranes were incubated with horseradish peroxidase (HRP)-conjugated goat anti-rabbit IgG antibody (1:3000; Abcam) for 2 h at room temperature and washed three times in TBS-T, and visualized with an ECL Plus kit.

### Statistical analysis

All statistical analyses were performed using SPSS 22.0 (SPSS, Chicago, IL). The gene expression level of lncRNA-LET in tumors was compared to that in adjacent normal tissues utilizing the Wilcoxon test, whereas the association between lncRNA-LET expression and clinical characteristics was evaluated using the chi-square test. Survival curves were plotted by the Kaplan-Meier method, and long-rank comparison was carried out to assess differences between stratified survival groups using the median value as the cut-off. A Cox proportional hazards analysis was performed to calculate the hazard ratio (HR) and the 95% confidence interval (CI) to evaluate the association between all covariates and progression free survival. All quantitative data were presented as Median ± Quartile range. Statistical differences were determined by a two-tailed *t* test in cell line experiments. Data were presented as mean ± standard deviation (mean ± SD). A *P* value of less than 0.05 was considered to be statistically significant.

## Abbreviations

NSCLC: non-small cell lung cancer
lncRNA: long non-coding RNA
lncRNA-LET: lncRNA low expression in tumor
(NICD1): Notch signaling, Notch1 intracellular domain
